# Clinical Trial Data Sharing for COVID-19–Related Research

**DOI:** 10.2196/26718

**Published:** 2021-03-12

**Authors:** Louis Dron, Alison Dillman, Michael J Zoratti, Jonas Haggstrom, Edward J Mills, Jay J H Park

**Affiliations:** 1 Cytel Canada Inc. Vancouver, BC Canada; 2 School of Public Health, Faculty of Medicine, Imperial College London London United Kingdom; 3 International COVID-19 Data Alliance London United Kingdom; 4 Department of Experimental Medicine, University of British Columbia Vancouver, BC Canada

**Keywords:** COVID-19, data-sharing, clinical trials, data, research, privacy, security, registry, feasibility, challenge, recruitment, error, bias, assessment, interoperability, dataset, intervention, cooperation

## Abstract

This paper aims to provide a perspective on data sharing practices in the context of the COVID-19 pandemic. The scientific community has made several important inroads in the fight against COVID-19, and there are over 2500 clinical trials registered globally. Within the context of the rapidly changing pandemic, we are seeing a large number of trials conducted without results being made available. It is likely that a plethora of trials have stopped early, not for statistical reasons but due to lack of feasibility. Trials stopped early for feasibility are, by definition, statistically underpowered and thereby prone to inconclusive findings. Statistical power is not necessarily linear with the total sample size, and even small reductions in patient numbers or events can have a substantial impact on the research outcomes. Given the profusion of clinical trials investigating identical or similar treatments across different geographical and clinical contexts, one must also consider that the likelihood of a substantial number of false-positive and false-negative trials, emerging with the increasing overall number of trials, adds to public perceptions of uncertainty. This issue is complicated further by the evolving nature of the pandemic, wherein baseline assumptions on control group risk factors used to develop sample size calculations are far more challenging than those in the case of well-documented diseases. The standard answer to these challenges during nonpandemic settings is to assess each trial for statistical power and risk-of-bias and then pool the reported aggregated results using meta-analytic approaches. This solution simply will not suffice for COVID-19. Even with random-effects meta-analysis models, it will be difficult to adjust for the heterogeneity of different trials with aggregated reported data alone, especially given the absence of common data standards and outcome measures. To date, several groups have proposed structures and partnerships for data sharing. As COVID-19 has forced reconsideration of policies, processes, and interests, this is the time to advance scientific cooperation and shift the clinical research enterprise toward a data-sharing culture to maximize our response in the service of public health.

The scientific community has made several important inroads in the fight against COVID-19. The pandemic has mobilized the global research community at an unparalleled scale [1-3]. From the start of the COVID-19 pandemic to date, 2516 clinical trials have been registered globally [4]. Most are within hospitalized patient contexts, with other trials focusing on outpatient treatment or prophylaxis, whether through vaccination or pre- or posttreatment prophylaxis. Of the 2516 registered clinical trials, records indicate that 1278 (50.79%) trials are still actively enrolling patients, 26 (1.03%) have suspended recruitment, 43 (1.70%) have terminated, and 67 (2.66%) have withdrawn [4]. However, it is important to note that the status of 28.22% (710/2516) of these trials have not been updated in their respective registries since they were first posted, whereas only 1.83% (46/2516) of the trials that are past their expected completion dates have reported results linked to their respective registries [4]. According to a living systematic review on randomized clinical trials for COVID-19 published in *The BMJ*, only 85 trials have been published as of October 21, 2020, despite the large number of trials that have been registered and reported as complete [5]. Of these 85 published trials, 54 (64%) trials reported information on planned sample size, and 25 (46%) did not meet their recruitment targets [5]. In fact, they recruited approximately half of their planned recruitment (median 52.3%; IQR 31.7%-80.6%) [5]. A summary of these findings is provided in Figure 1.

**Figure 1:**
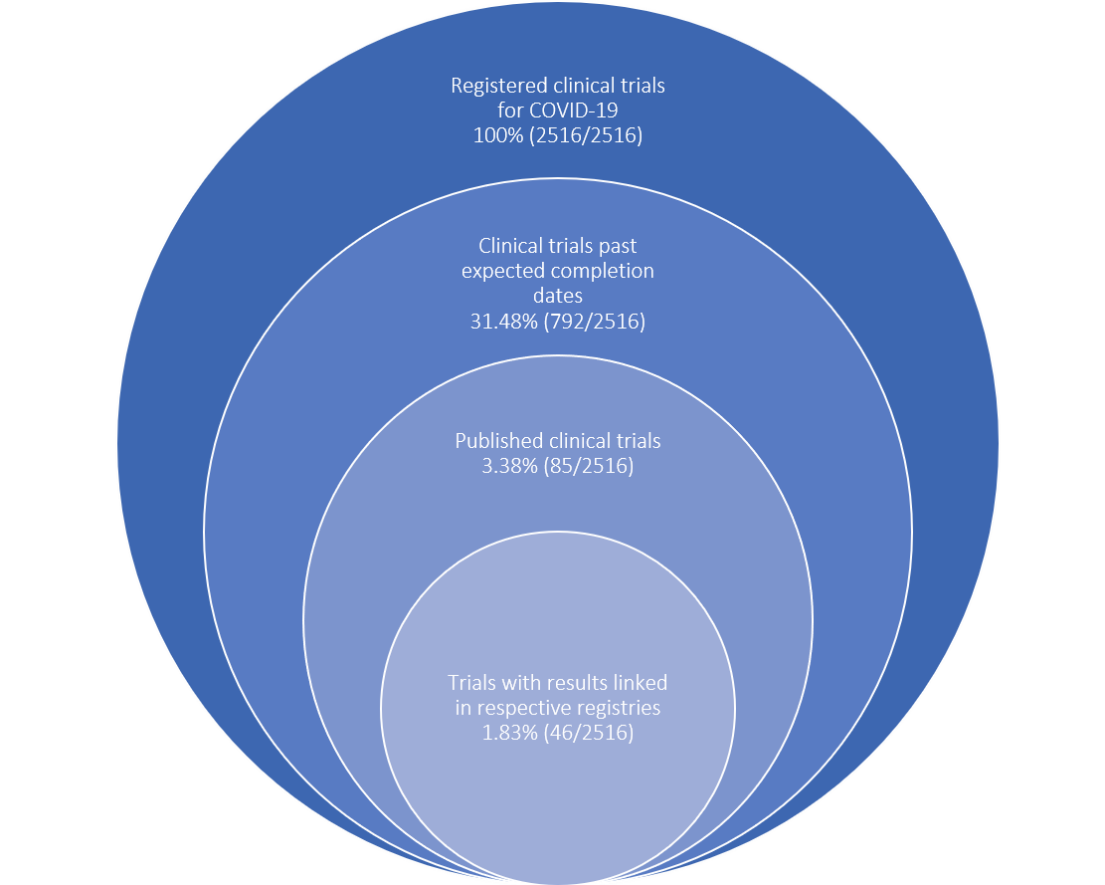
Summary of registered clinical trial status for COVID-19–related research.

During this time, we have seen a large number of trials conducted without results being made available. It is likely that a plethora of COVID-19 trials have stopped early, not for statistical reasons but due to lack of feasibility [4]. Reasons for studies becoming nonfeasible are extensive, ranging from unwillingness to participate due to quarantine, challenges in telemedicine solutions for trials [6], and emergency changes to staff resourcing [7]. Furthermore, there are likely feasibility challenges in the recruitment dependent on the patient context. Trials range from patients in intensive care to healthy volunteers in vaccine and treatment prophylaxis trials. As such, contributions of recruitment competition and patient hesitancy to the lack of feasibility are heavily treatment-context driven. Accordingly, many clinical trials during the COVID-19 pandemic have faced a multitude of challenges related to consenting and recruiting new participants given the proliferation of trials that are competing for recruiting eligible participants into their own respective trials [8,9].

Trials stopped early for feasibility are, by definition, statistically underpowered and thereby prone to inconclusive findings. Statistical power is not necessarily linear with the total sample size, and even small reductions in patient numbers or events can have a substantial impact on the research outcomes. Given the profusion of clinical trials investigating identical or similar treatments across different geographical and clinical contexts, one must also consider that the likelihood of the substantial numbers of false-positive and false-negative trials, emerging with the increasing overall number of trials, adds to public perceptions of uncertainty. Complicating this issue is the evolving nature of the pandemic, where baseline assumptions on control group risk factors used to develop sample size calculations are far more challenging than those in the case of well-documented diseases.

The standard answer to address these challenges during nonpandemic settings is to rigorously assess each trial for statistical power and risk-of-bias and then pool the reported aggregated results using meta-analytic statistical approaches. This solution simply will not suffice for COVID-19. Even with random-effects meta-analysis models, it will be difficult to adjust for heterogeneity of different trials with aggregated reported data alone, especially given the absence of common data standards and outcome measures. Common data standards are a key feature of system interoperability and facilitate synthesis methodologies to be rapidly scaled, such as meta-analyses. To date, several groups have proposed structures and partnerships for data sharing in the context of COVID-19, some of which are integrated with prespecified statistical analysis methodologies [10,11]. Given the substantial under-recruitment reported to date, vast numbers of trials will be underpowered. This lack of power may be due to design challenges, or a consequence of termination prior to reaching the recruitment target. As such, integration of different trial datasets for individual participant-level data (IPD) meta-analyses may be the only solution in determining what works and is safe for COVID-19. In an IPD meta-analysis, rather than measuring aggregate study-level outputs, data can be taken from either all or a proportion of participants within individual studies. In doing so, more nuanced comparisons between patient groups is possible. For example, participants across two trials may have, on average, significant differences in demographics to one another, yet substantial proportions of patients across both trials may have sufficient similarity for a valid analysis. Meta-analyses integrating IPD have a number of potential methodological advantages, particularly when subpopulations of interest demonstrate promising treatment signals. In particular, IPD meta-analyses allow for more effective subgroup analyses and better statistical power for detecting treatment interaction effects [12] in cases wherein differences between populations are marked. These methods are endorsed by the Cochrane collaboration—a useful tool, especially when treatment effects are influenced by the follow-up duration. As COVID-19 research evolves to longer-term outcomes (sometimes referred to as “long-COVID”) [13], these analytical advantages are likely to develop further. The key to the process of evidence synthesis is the appropriate selection of trials with comparable patient populations and design features, such as outcome definitions [14]. In the absence of unified data structures and data sharing agreements, this process may either be time consuming or entirely nonfeasible, depending on data heterogeneity.

To serve the public who are waiting for the medical research community to efficiently make medical discoveries, the COVID-19 pandemic has (arguably) mandated sharing IPD into a public health obligation. Although the International Committee of Journal Editors has previously discussed the importance of data sharing of clinical trials, this discussion has largely been limited to published clinical trials [15]. The data sharing mandate for the COVID-19 pandemic should be extended to all clinical trials, including those trials that will not be published because they ended early for feasibility reasons. Other informal data sharing platforms for ongoing trials are available, such as clinical trial registries, which can provide information on outcome measures and broad design features [4].

Sharing of IPD has historically proven to be challenging, as investigators and sponsors have held tight to their data for academic, regulatory, and commercial reasons. However, the health and economic consequences of the pandemic thus far signal a need to mandate data sharing, expedite systems to apportion credit for data sharing, and preserve commercial interests. The need to share and collaborate openly supersedes our personal career or organizational goals. This sentiment has been shared among the research community, and many organizations (such as the Wellcome trust [16]) have quickly identified the need to share data more rapidly than has historically been the case.

The International COVID-19 Data Alliance (ICODA) provides an example of recent improvements in data sharing within the context of a global pandemic [17,18]. Convened by Health Data Research UK, ICODA is an international health data-led research response that seeks to provide a platform to enable researchers to access global data to derive insights about COVID-19 to advance the development of therapeutics [17,18]. The organization recognizes the urgent need to enable access to data that can be linked with other data in a safe and secure way.

As the processes for addressing personal privacy, data security, and data standardization have become sufficiently more sophisticated in recent years, barriers previously considered to be insurmountable have been minimized [10,19]. Investigators that have launched clinical trials can utilize existing global clinical research data sharing platforms such as Vivli [19], TransCelerate Biopharma [20], and ICODA [17,18], to collectively and securely curate and analyze their findings. Taken together, data from different trials can answer meaningful public health questions while avoiding the risk of becoming inconclusive in isolation. Investigators are keen for data; as represented by Vivli [11], as of December 2020, over 200 requests for trial-level data have been made in 2020, although no publications utilizing COVID-19 data are available from this group at present. Challenges in execution of these methods and strategies are multifaceted, involving researcher awareness of resource availability, technical capacity for analysis, and access agreements from data providers. To this end, we applaud the efforts of groups mentioned above in reaching out to researchers for proposals and taking strides toward simplifying the often challenging process of providing patient-level data. Successful analysis and subsequent publications utilizing these methods may provide an informative case study to promote further researcher contribution.

Particularly in the context of a pandemic, researchers, policymakers, and the general public are finding challenges in navigating the multitudes of data available daily. In tandem, high-profile instances of retractions owing to poor data screening [21] have led many to reach “data fatigue” [22]. Here, data synthesis exercises that utilize the aforementioned statistical efficiencies of patient-level data provide an avenue through which data fatigue may be minimized and succinct summaries that may otherwise be unachievable, as well as improve awareness of therapeutic trends in COVID-19.

We hope that the COVID-19 pandemic is a historic turning point of a sharing culture in the medical research community. The need for rapid and robust clinical research for the discovery of effective and safe therapeutics and vaccines has never been higher. Strengthening our public health response to COVID-19 will require larger collated patient-level datasets to facilitate the scientific precision required for answers on COVID-19 medical interventions. As COVID-19 has forced reconsideration of policies, processes, and interests, this is the time to advance scientific cooperation and shift the clinical research enterprise toward a data-sharing culture that can maximize our response in the service of public health.

## Abbreviations

ICODA: International COVID-19 Data Alliance
IPD: individual participant-level data
